# Genome sequencing, annotation and comparative genomic analysis of *Shigella dysenteriae* strain SD1D

**DOI:** 10.1186/1757-4749-6-28

**Published:** 2014-07-11

**Authors:** Gurwinder Kaur, Sathyaseelan Sathyabama, Amit Arora, Sheenam Verma, Nida Mubin, Javed N Agrewala, Shanmugam Mayilraj

**Affiliations:** 1 Microbial Type Culture Collection and Gene bank, CSIR-Institute of Microbial Technology, Sector 39A, Chandigarh 160036, India; 2 Immunology Laboratory, CSIR-Institute of Microbial Technology, Sector 39A, Chandigarh 160036, India

**Keywords:** *Shigella dysenteriae*, Shigellosis, G + C content, EzTaxon, RAST

## Abstract

**Background:**

Shigellosis is an acute form of gastroenteritis caused by the bacteria belonging to the genus *Shigella*. It is the most common cause of morbidity and mortality in children. *Shigella* belongs to the family *Enterobactericeae*, which is a Gram-negative and rod shaped bacterium. In the present study, we report the draft genome of *Shigella dysenteriae* strain SD1D, which was isolated from the stool sample of a healthy individual.

**Results:**

Based on 16S rRNA gene sequence and phylogenetic analysis, the strain SD1D was identified as *Shigella dysenteriae*. The draft genome of SD1D consisted of 45, 93, 159 bp with a G + C content of 50.7%, 4, 960 predicted CDSs, 75 tRNAs and 2 rRNAs. The final assembly contained 146 contigs of total length 45, 93, 159 bp with N_50_ contig length of 77, 053 bp; the largest contig assembled measured 3, 85, 550 bp.

**Conclusions:**

We have for the first time performed the whole genome sequencing of *Shigella dysenteriae* strain SD1D. The comparative genomic analysis revealed several genes responsible for the pathogenesis, virulence, defense, resistance to antibiotics and toxic compounds, multidrug resistance efflux pumps and other genomic features of the bacterium.

## Background

The genus *Shigella* was first proposed by Shiga in 1898 [1] and later on emended by Castellani and Chalmers in 1919 [2]. At present, the genus *Shigella* consists of four recognized species *Shigella dysenteriae*[2], *Shigella boydii*[3], *Shigella flexneri*[2] and *Shigella sonnei*[4]. *Shigella dysenteriae* is the type species of the genus *Shigella*. Shigellosis is caused by any of the four above mentioned species of *Shigella*. Shigellosis is a form of acute gastroenteritis, involving inflammation in the gastrointestinal tract resulting in vomiting, abdominal pain, diarrhea and cramping. The virulence associated with *S. dysenteriae* is due to the production of an exotoxin called Shiga toxin (Stx), which is not excreted by the microorganism, but is released only during cell lysis [5]. Identification of *Shigella* species is important because of its role in diseases with particular reference to epidemics. The current gold standard for the detection of *Shigella* species in fecal specimens involves isolation, growth and identification of *Shigella* in the cultures. Isolates of *Shigella* can also be identified using serological tests [6]. Understanding the antibiotic resistance patterns of *Shigellae* and molecular characterization of plasmids and other genetic elements are also epidemiologically useful. All the four species of the genus *Shigella* have been whole genome sequenced: *Shigella boydii* (02 isolates) strain BS512*, Shigella dysenteriae* (01 isolate) strain M131649, *Shigella flexneri* 2a (04 isolates) strain 2457T and *Shigella sonnei* (02 isolates) strain 53G. For the first time we have performed whole genome sequencing, assembly and annotation of strain *Shigella dysenteriae* SD1D, which was isolated from the stool sample of healthy individual. In order to understand the correlation between *Shigella dysenteriae* strain SD1D and *Shigella spp.*, it is imperative to explore the genome of the strain SD1D and perform a comparative genomic analysis with *Shigella spp.* This would unveil the pathogenic potential of this strain in healthy individuals. In the current study, the complete genome sequence of *Shigella dysenteriae* strain SD1D and a functional level genomic comparison with *Shigella dysenteriae* strain M131649, *Shigella sonnei* strain 53G, *Shigella flexneri* 2a strain 2457T and *Shigella boydii* strain BS512 genomes was accomplished. The results suggest possible differences in the genes involved in virulence, disease and defense; phages and prophages among the species of the genus *Shigella.*

## Methods

### Isolation, identification, DNA extraction, genome sequencing, assembly and annotation

*Shigella dysenteriae* strain SD1D was isolated from stool sample of a healthy individual on March 19, 2013, using tryptic soya agar (TSA, HiMedia, India). *Shigella dysenteriae* strain SD1D was identified by 16S rRNA gene sequencing (1487 bp). Genomic DNA was extracted and amplification was performed using primers 27f (5′-AGAGTTTGATCCTGGCTCAG-3′) and 1500r (5′-AGAAAGGAGGTGATCCAGGC-3′). Agarose gel (1%) electrophoresis was used to separate amplified PCR fragment, which was subjected to gel elution and purification using QIAquick gel extraction kit (Qiagen). Further, four forward and three reverse primers were used for sequencing the purified PCR product. These were 27f (5′-AGAGTTTGATCCTGGCTCAG-3′), 357f (5′-CTCCTACGGGAGGCAGCAG-3′), 704f (5′-TAGCGGTGAAATGCGTAGA- 3′), 1114f (5′-GCAACGAGCGCAACC-3′), 685r (5′-TCTACGCATTTCACCGCTAC-3′), 1110r (5′-GGGTTGCGCTCGTTG-3′) and 1500r (5′-GAAAGGAGGTGATCCAGGC-3′) (*Escherichia coli* numbering system) [7]. Identification of phylogenetic neighbors and the calculation of pairwise 16S rRNA gene sequence similarities were achieved using the EzTaxon server [8] and aligned using MEGA version 5.0 [9]. Phylogenetic trees were constructed using the neighbor-joining as well as maximum parsimony algorithms. Bootstrap analysis was performed to evaluate the confidence limits of the branching. The genome of *Shigella dysenteriae* strain SD1D was sequenced using a standard run of Illumina HiSeq 1000 sequencing technology at c-CAMP, next generation genomic facility, Bengaluru, India (http://www.ccamp.res.in), which produced a total of 29, 186, 504 paired-end reads (paired distance (insert size) ~330 bp) of 101 bp. CLC Bio Workbench v6.0.4 (CLC Bio, Denmark) was employed for pre-processing the data to trim and remove low quality sequences. A total of 2, 90, 47, 554 high quality, vector filtered reads ~568X were used for assembly with CLC Bio Workbench (at word size of 45 and bubble size of 98. Function based comparative genomic analysis for *Shigella dysenteriae* strain SD1D, *Shigella dysenteriae* strain M131649, *Shigella sonnei* strain 53G, *Shigella flexneri* 2a strain 2457T and *Shigella boydii* strain BS512 was performed with the help of RAST (Rapid Annotation using Subsystem Technology) system. Final genome draft was employed for genome annotation using RAST server and RNAmmer 1.2 server [10,11].

## Quality assurance

Based on the 16S rRNA gene sequence, phylogenetic analysis, morphological and biochemical characterization, the strain SD1D was identified as *Shigella dysenteriae*. Cells of strain SD1D were Gram-negative rods, facultative anaerobes; positive for uitilization of D-fructose, L-rhamnose, sodium citrate, maltose, D-sorbitol and negative for adonitol, cellobiose, gentibiose and raffinose; positive for oxidase production and tween 80 hydrolysis.

**Figure 1:**
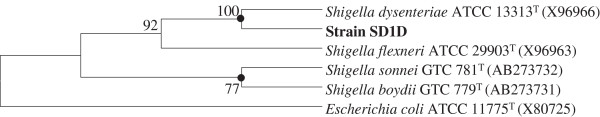
**Phylogenetic tree using ‘neighbor-joining’ algorithm on 16S rRNA gene sequences showing the relationship between *Shigella dysenteriae *strain SD1D and related members of the genus *Shigella.****Escherichia coli* ATCC 11775^T^ (X80725) was used as an out-group. Bootstrap values (expressed as percentages of 100 replications) greater than 50% are given at nodes. Filled circles indicate that corresponding nodes were also recovered in the tree constructed with maximum parsimony and maximum likelihood. GenBank accession numbers are given in parentheses.

To assess the purity of strain SD1D, the 16S rRNA gene sequence of strain SD1D was aligned with sequences of other members of genus *Shigella* retrieved from EzTaxon data base. The strain SD1D showed highest degree of similarity with *Shigella dysenteriae* strain ATCC 13313^T^ (100%) followed by *Shigella flexneri* strain ATCC 29903^T^ (99.13%), *Shigella sonnei* strain GTC 781^T^ (98.98%) and *Shigella boydii* strain GTC 779^T^ (98.58%). Also the phylogenetic analyses using neighbor-joining, maximum parsimony and maximum likelihood algorithm revealed that the strain SD1D formed a separate branch within the lineage that included *Shigella dysenteriae* (Figure 1).

## Initial findings

### Genomic features

**Figure 2:**
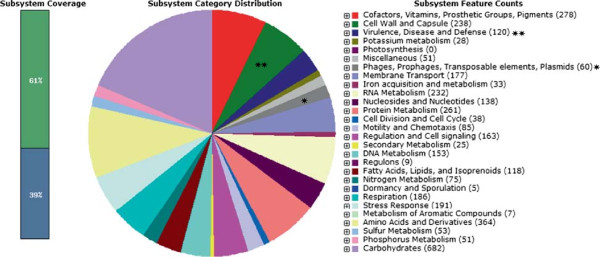
**Sub-system distribution of *Shigella dysenteriae *** **strain SD1D (based on RAST annotation server).**

**Figure 3:**
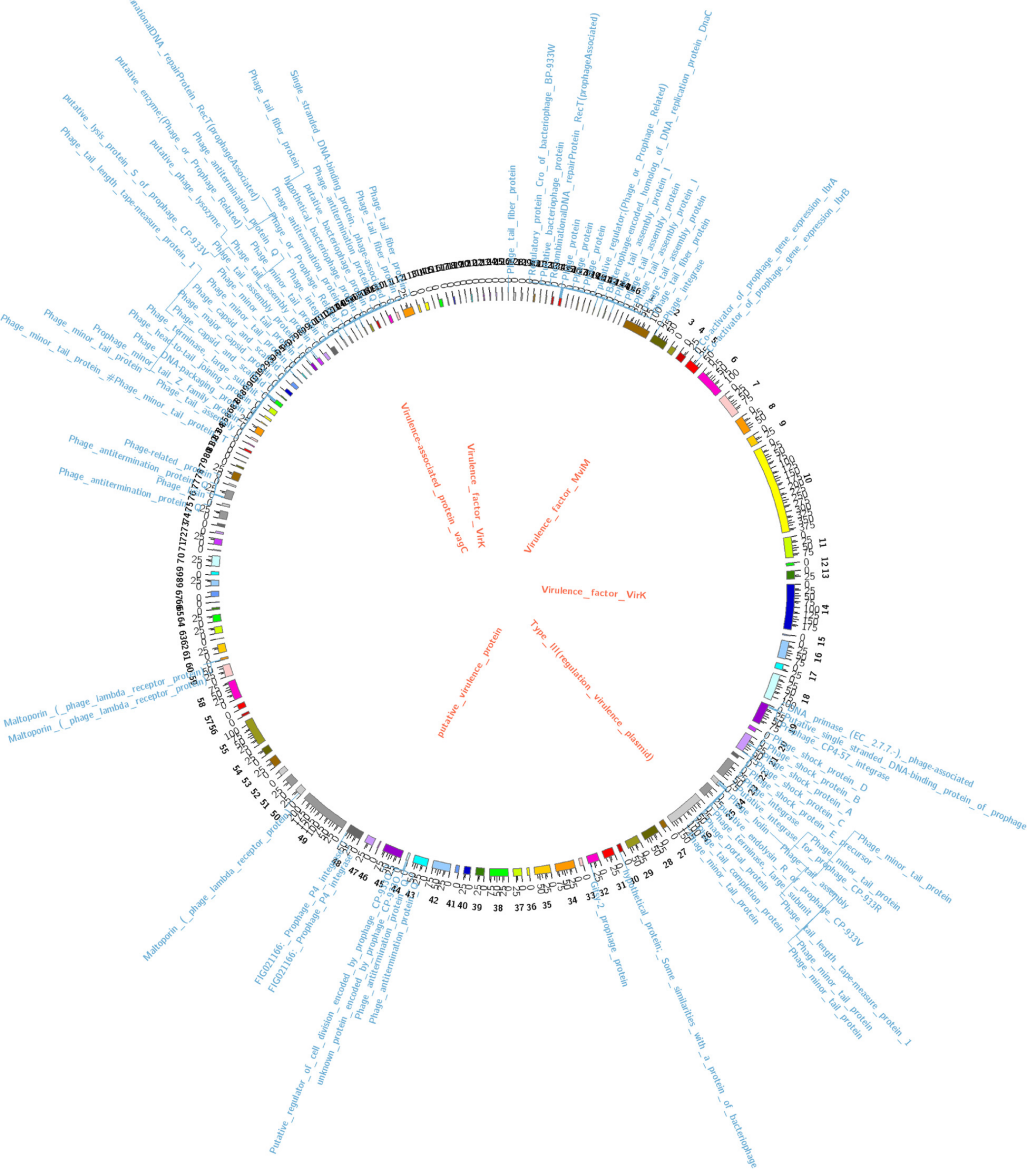
**Circular genome map of *Shigella dysenteriae *strain SD1D showing the major genes and their regulators.** The 146 assembled contigs are shown by different colored ideograms having their base-pair positions depicted at a scale of 1000 units. The coverage of the assembly at each base pair can be seen by the grey color track. Annotations descriptors for “virulence” genes [inner label: red] and “phage” related genes [outer label: blue] are mapped onto their respective contig positions. Many annotation descriptors occupy neighboring positions on the contigs; the descriptors are stacked to allow better visualization.

The genome size of *Shigella dysentriae* strain SD1D consisted of 45, 93, 159 bp. The G + C content was 50.7% with 4,960 CDSs, 75 tRNAs and 2 rRNAs. Among this, 45, 93, 159 bp were identified and 146 contigs were predicted. The largest contig consisted of 3, 85, 550 bp and the length of N_50_ contig was 77, 053 bp. Number of subsystems were 582. Summary of the basic features of the genome is given in Table 1. Sub-system distribution of *Shigella dysenteriae* strain SD1D is depicted in Figure 2 (based on RAST annotation server). The graphical circular map of the genome is represented in Figure 3.

**Table 1:** **Summary of the annotated genome of *Shigella dysenteriae *** **strain SD1D**

**Characters**	**Length (bp)**		
N_75_	37, 003		
N_50_	77, 053		
N_25_	1, 16, 745		
Minimum	1,031		
Maximum	3, 85, 550		
Average	31,460		
Count	146		
Total	45, 93,159		
**Nucleotide**	**Count**	**Frequency (%)**	**G + C mol %**
Adenine (A)	1, 131, 809	24.6	
Cytosine (C)	1, 166, 743	25.4	50.7
Guanine (G)	1, 161, 758	25.3	
Thymine (T)	1, 131, 369	24.6	
Any nucleotide (N)	1, 480	0.0	

### Function based comparative genomic analysis

In strain *Shigella dysenteriae* SD1D, we identified a total of 3, 335 genes and assessed the presence of 66 protein coding genes involved in various functions. These included genes for virulence, disease and defense, phages, prophages, transposable elements, and plasmids (Additional file 1: Table S1). It was observed that out of 66 genes, 37 genes encoded for functional proteins exclusively in *Shigella dysenteriae* strain SD1D and attributed to pathogenicity. There are 22 genes that are responsible for virulence, disease and defense and code for Accessory colonization factor; AcfD precursor; Uncharacterized protein YidS; Cation efflux system protein CusC precursor; Cation efflux system protein CusF precursor; Cobalt-Zinc-Cadmium efflux RND transporter membrane fusion protein CzxB family; Copper sensory histidine kinase CusS; Copper sensing two component system response regulator CusR; Heavy metal sensor histidine kinase; CopG protein; Copper resistance protein B; Copper resistance protein C precursor; Multi copper oxidase; PF00070 family FAD-dependent NAD(P)-disulphide oxidoreductase; Inner membrane component of tripartite multi drug resistance system; Outer membrane component of tripartite multi drug resistance system; Multi drug efflux transporter, major facilitator super family (MFS); Multiple antibiotic resistance protein MarB; Multi drug transporter MdtB; Multi drug transporter MdtC; Probable RND efflux membrane fusion protein; Beta lactamase class C and other penicillin binding proteins; Metal dependent hydrolases of the beta-lactamase super family I. Also 15 genes encoded for phages, prophages, transposable elements, plasmids that confer horizontal gene transfer. These include Phage capsid and scaffold; Phage major capsid protein; Phage portal protein; Integron integrase IntI1; Single stranded DNA-binding protein, phage-associated; Phage tail fiber proteins; Phage minor tail protein; Phage tail assembly; Phage tail assembly protein; Phage tail completion protein; Phage tail assembly protein I; Phage tail length tape measure protein 1; Co-activator of prophage gene expression IbrA, IbrB.

Apart from the above mentioned genes there are 29 genes that are absent in strain SD1D but are present in *Shigella dysenteriae* strain M131649, *Shigella sonnei* strain 53G, *Shigella flexneri* 2a strain 2457T and *Shigella boydii* strain BS512. These involve genes coding for Translation elongation factor Tu; DNA binding heavy metal response regulator; Cytoplasmic copper homeostasis protein CutC L-cystine ABC transporter periplasmic cystine binding protein; Spectinomycin 9-O-adenyltransferase; Streptomycin 3″-O- adenyltransferase; Anion permease ArsB/Nha D-like; Arsenic resistance protein ArsH; Arsenic resistance protein ACR3; Cyctoplasmic copper homeostasis protein CutC; Mercuric resistance operon coregulator; Mercuric resistance operon regulatory protein; Mercuric transport protein MerC; Mercuric transport protein MerT; Periplasmic mercury (+2) binding protein; Membrane fusion protein of RND family multi drug efflux pump RND efflux system, membrane fusion protein CmeA; Colicin E2 tolerance protein CbrC; Capsid scaffolding protein; Phage capsid protein; Phage head completion stabilization protein; Phage head maturation protease; Phage terminase ATPase subunit; Phage terminase, endonuclease subunit; Phage terminase, small subunit; ISPsy4, transposition helper protein; TniA putative transposase; TniB NTP-binding protein; Transposase OrfAB, subunit B. Remarkably, it was observed that genes for arsenic resistance was present in all the strains of *Shigella* mentioned above, and not in strain SD1D.

## Future directions

Genome analysis in *S. dysentriae* strain SD1D provides extensive information regarding the identification of traits (genes involved in antibiotic resistance, horizontal gene transfer etc.) responsible for host pathogen interaction, which could be harnessed for developing new drugs and vaccines. As it has been noticed in the case of malaria parasite, where the whole genome is exploited to develop and design new anti-malarial drug targets [12]. There are also reports that prove that there is a frequent acquisition of antibiotic resistance genes in *S. dysenteriae* Sd1, which contributed to virulence [13]. Metabolic pathway analysis showed that *S. dysenteriae* strain SD1D has high tendency to become pathogenic due to acquisition of antibiotic resistance genes from external sources. Some of the genes conferring resistance in strain SD1D are copper resistance protein C precursor, outer and inner components of tripartite multidrug resistance system etc. These pathways could be analyzed in the future for identifying possible drug targets and vaccine candidates.

## Conclusion

We have for the first time sequenced the whole genome of *Shigella dysenteriae* strain SD1D that was isolated from stool sample of a healthy individual. Further, genomic analysis revealed the genes responsible for the pathogenesis, virulence, defense, resistance to antibiotics and toxic compounds, multidrug resistance efflux pumps and other genomic features of the bacterium. The genome of strain SD1D consisted of 45, 93, 159 bp with a G + C content of 50.7%, 4, 960 predicted CDSs and 75 tRNAs and 2 rRNAs. Genome mining and research on the genome of *Shigella dysenteriae* strain SD1D may reveal the potential cause for its pathogenicity and virulence.

### Ethical clearance

The study was ethically approved by the Institutional Biosafety Committee of the Institute of Microbial Technology (Ref/IBSC/2012-2/09).

## Availability of supporting data

This Whole Genome Shotgun project has been deposited at DDBJ/EMBL/GenBank under the accession AURX00000000. The version described in this paper is the first version, AURX01000000.

## Competing interests

The author’s declare that they have no competing interests.

## Authors’ contributions

Performed experiments: GK, SS, AA, SV and NM. Planned and executed experiments analyzed data and wrote manuscript: JNA and SM. All authors read and approved the final manuscript.

## Supplementary Material

Additional file 1: Table S1 Function based (Virulence, Disease and Defense and Phages, Prophages, Transposable elements, Plasmids) comparative genomic analysis of strains (1) *Shigella dysenteriae* strain SD1D, 2. *Shigella dysenteriae* strain M 131649, 3. *Shigella sonnei* strain 53G, 4. *Shigella flexneri* strain 2a 2457T and 5. *Shigella boydii* strain BS 512. Click here for file
