# Erratum to: Genome sequencing, annotation and comparative genomic analysis of *Shigella dysenteriae* strain SD1D

**DOI:** 10.1186/s13099-016-0120-6

**Published:** 2016-08-10

**Authors:** Gurwinder Kaur, Sathyaseelan Sathyabama, Amit Arora, Sheenam Verma, Nida Mubin, Javed N. Agrewala, Shanmugam Mayilraj

**Affiliations:** 1 Microbial Type Culture Collection and Gene Bank, CSIR-Institute of Microbial Technology, Sector 39A, Chandigarh, 160036 India; 2 Immunology Laboratory, CSIR-Institute of Microbial Technology, Sector 39A, Chandigarh, 160036 India

## Erratum to: Gut Pathog (2014) 6:28 DOI 10.1186/1757-4749-6-28

Following publication of the article in *Gut Pathogens* [1], it was brought to our attention that the isolation date mentioned in the Methods section is incorrect and should read October 9, 2012 instead of March 19, 2013. The full sentence should read:

“*Shigella dysenteriae* strain SD1D was isolated from stool sample of a healthy individual on Oct 9, 2012, using tryptic soya agar (TSA, HiMedia, India).”
