# Supplementary material for: Genome sequencing, annotation and comparative genomic analysis of Shigella dysenteriae strain SD1D
Source: Gut Pathog. 2014 Jul 11;6:28. doi: 10.1186/1757-4749-6-28 (PMC4099087; doi:10.1186/1757-4749-6-28)
Supplement: Additional file 1: Table S1 — Function based (Virulence, Disease and Defense and Phages, Prophages, Transposable elements, Plasmids) comparative genomic analysis of strains (1) Shigella dysenteriae strain SD1D, 2. Shigella dysenteriae strain M 131649, 3. Shigella sonnei strain 53G, 4. Shigella flexneri strain 2a 2457T and 5. Shigella boydii strain BS 512. [file 1757-4749-6-28-S1.doc]

**Additional file**

**Table S1. Function based (Virulence, Disease and Defence and Phages, Prophages, Transposable elements, Plasmids) comparative genomic analysis of strains (1) Shigella dysenteriae strain SD1D, 2. Shigella dysenteriae strain M 131649, 3. Shigella sonnei strain 53G, 4. Shigella flexneri strain 2a 2457T and 5. Shigella boydii strain BS 512.**

| **Major Genes** | **Major Functional features** | **Characteristic genes** | **Function** | **1** | **2** | **3** | **4** | **5** |
| --- | --- | --- | --- | --- | --- | --- | --- | --- |
| Virulence, Disease and Defense | Adhesion | Accessory colonization factor | Accessory colonization factor AcfD precursor | Yes | No | No | No | No |
| Virulence, Disease and Defense | Adhesion | Mediator of hyperadherence YidE in Enterobacteria and its conserved | Uncharacterized protein YidS | Yes | No | No | No | No |
| Virulence, Disease and Defense | Invasion and intracellular resistance | Mycobacterium virulence operon involved in protein synthesis | Translation elongation factor Tu | No | Yes | Yes | Yes | Yes |
| Virulence, Disease and Defense | Resistance to antibiotics and toxic compounds | Cobalt-Zinc-Cadmium resistance | Cation efflux system protein CusC presursor | Yes | No | No | No | No |
| Virulence, Disease and Defense | Resistance to antibiotics and toxic compounds | Cobalt-Zinc-Cadmium resistance | Cation efflux system protein CusF presursor | Yes | No | No | No | No |
| Virulence, Disease and Defense | Resistance to antibiotics and toxic compounds | Cobalt-Zinc-Cadmium resistance | Cobalt-Zinc-Cadmium efflux RND transporter membrane fusion protein CzxB family | Yes | No | No | No | No |
| Virulence, Disease and Defense | Resistance to antibiotics and toxic compounds | Cobalt-Zinc-Cadmium resistance | Copper sensory histidine kinase CusS | Yes | No | No | No | No |
| Virulence, Disease and Defense | Resistance to antibiotics and toxic compounds | Cobalt-Zinc-Cadmium resistance | Copper sensing two component system response regulator CusR | Yes | No | No | No | No |
| Virulence, Disease and Defense | Resistance to antibiotics and toxic compounds | Cobalt-Zinc-Cadmium resistance | DNA binding heavy metal response regulator | No | Yes | Yes | Yes | Yes |
| Virulence, Disease and Defense | Resistance to antibiotics and toxic compounds | Cobalt-Zinc-Cadmium resistance | Heavy metal sensor histidine kinase | Yes | No | No | No | No |
| Virulence, Disease and Defense | Resistance to antibiotics and toxic compounds | Copper homeostasis | CopG protein | Yes | No | No | No | No |
| Virulence, Disease and Defense | Resistance to antibiotics and toxic compounds | Copper homeostasis | Copper resistance protein B | Yes | No | No | No | No |
| Virulence, Disease and Defense | Resistance to antibiotics and toxic compounds | Copper homeostasis | Copper resistance protein C precursor | Yes | No | No | No | No |
| Virulence, Disease and Defense | Resistance to antibiotics and toxic compounds | Copper homeostasis | Multi copper oxidase | Yes | No | No | No | No |
| Virulence, Disease and Defense | Resistance to antibiotics and toxic compounds | Copper homeostasis: copper tolerance | Cytoplasmic copper homeostasis protein CutC | No | Yes | Yes | Yes | Yes |
| Virulence, Disease and Defense | Resistance to antibiotics and toxic compounds | Mercuric reductase | PF00070 family FAD-dependent NAD(P)- disulphide oxireductase | Yes | No | No | No | No |
| Virulence, Disease and Defense | Resistance to antibiotics and toxic compounds | Adaption to d-cysteine | L-cystine ABC transporter periplasmic cystine binding protein | No | Yes | Yes | Yes | Yes |
| Virulence, Disease and Defense | Resistance to antibiotics and toxic compounds | Multi drug resistance, Tripartite Systems found in Gram negative bacteria | Inner membrane component of tripartite multi drug resistance system | Yes | No | No | No | No |
| Virulence, Disease and Defense | Resistance to antibiotics and toxic compounds | Multi drug resistance, Tripartite Systems found in Gram negative bacteria | Outer membrane component of tripartite multi drug resistance system | Yes | No | No | No | No |
| Virulence, Disease and Defense | Resistance to antibiotics and toxic compounds | Multi drug resistance efflux pumps | Multi drug efflux transporter, major facilitator super family (MFS) | Yes | No | No | No | No |
| Virulence, Disease and Defense | Resistance to antibiotics and toxic compounds | Multiple antibiotic resistance MAR locus | Multiple antibiotic resistance protein MarB | Yes | No | No | No | No |
| Virulence, Disease and Defense | Resistance to antibiotics and toxic compounds | The mdt ABCD multi drug resistance cluster | Multi drug transporter MdtB | Yes | No | No | No | No |
| Virulence, Disease and Defense | Resistance to antibiotics and toxic compounds | The mdt ABCD multi drug resistance cluster | Multi drug transporter MdtC | Yes | No | No | No | No |
| Virulence, Disease and Defense | Resistance to antibiotics and toxic compounds | The mdt ABCD multi drug resistance cluster | Probable RND efflux membrane fusion protein | Yes | No | No | No | No |
| Virulence, Disease and Defense | Resistance to antibiotics and toxic compounds | Aminoglycoside adenyl transferases | Spectinomycin 9-O-adenyltransferase | No | Yes | Yes | Yes | Yes |
| Virulence, Disease and Defense | Resistance to antibiotics and toxic compounds | Aminoglycoside adenyl transferases | Streptomycin 3”-O-adenyltransferase | No | Yes | Yes | Yes | Yes |
| Virulence, Disease and Defense | Resistance to antibiotics and toxic compounds | Arsenic resistance | Anion permease ArsB/Nha D-like | No | Yes | Yes | Yes | Yes |
| Virulence, Disease and Defense | Resistance to antibiotics and toxic compounds | Arsenic resistance | Arsenic resistance protein ArsH | No | Yes | Yes | Yes | Yes |
| Virulence, Disease and Defense | Resistance to antibiotics and toxic compounds | Arsenic resistance | Arsenic resistance protein ACR3 | No | Yes | Yes | Yes | Yes |
| Virulence, Disease and Defense | Resistance to antibiotics and toxic compounds | Copper homeostasis: copper tolerance | Cyctoplasmic copper homeostasis protein CutC | No | Yes | Yes | Yes | Yes |
| Virulence, Disease and Defense | Resistance to antibiotics and toxic compounds | Mercury resistance operon | Mercuric resistance operon coregulator | No | Yes | Yes | Yes | Yes |
| Virulence, Disease and Defense | Resistance to antibiotics and toxic compounds | Mercury resistance operon | Mercuric resistance operon regulatory protein | No | Yes | Yes | Yes | Yes |
| Virulence, Disease and Defense | Resistance to antibiotics and toxic compounds | Mercury resistance operon | Mercuric transport protein MerC | No | Yes | Yes | Yes | Yes |
| Virulence, Disease and Defense | Resistance to antibiotics and toxic compounds | Mercury resistance operon | Mercuric transport protein MerT | No | Yes | Yes | Yes | Yes |
| Virulence, Disease and Defense | Resistance to antibiotics and toxic compounds | Mercury resistance operon | Periplasmic mercury (+2) binding protein | No | Yes | Yes | Yes | Yes |
| Virulence, Disease and Defense | Resistance to antibiotics and toxic compounds | Multi drug resistance efflux pumps | Membrane fusion protein of RND family multi drug efflux pump | No | Yes | Yes | Yes | Yes |
| Virulence, Disease and Defense | Resistance to antibiotics and toxic compounds | Multi drug resistance efflux pumps | RND efflux system, membrane fusion protein CmeA | No | Yes | Yes | Yes | Yes |
| Virulence, Disease and Defense | Resistance to antibiotics and toxic compounds | Beta-lactamase | Beta lactamase class C and other penicillin binding proteins | Yes | No | No | No | No |
| Virulence, Disease and Defense | Resistance to antibiotics and toxic compounds | Beta-lactamase | Metal dependent hydrolases of the beta-lactamase super family I | Yes | No | No | No | No |
| Virulence, Disease and Defense | Bacteriocins, ribosomally synthesized antibacterial | Tolerance to colicin E2 | Colicin E2 tolerance protein CbrC | No | Yes | Yes | Yes | Yes |
| Phages, Prophages, Transposable elements, Plasmids | Phages, Prophages | Phage capsid proteins | Phage capsid and scaffold | Yes | No | No | No | No |
| Phages, Prophages, Transposable elements, Plasmids | Phages, Prophages | Phage capsid proteins | Phage major capsid protein | Yes | No | No | No | No |
| Phages, Prophages, Transposable elements, Plasmids | Phages, Prophages | Phage capsid proteins | Capsid scaffolding protein | No | Yes | Yes | Yes | Yes |
| Phages, Prophages, Transposable elements, Plasmids | Phages, Prophages | Phage capsid proteins | Phage capsid protein | No | Yes | Yes | Yes | Yes |
| Phages, Prophages, Transposable elements, Plasmids | Phages, Prophages | Phage capsid proteins | Phage head completion stabilization protein | No | Yes | Yes | Yes | Yes |
| Phages, Prophages, Transposable elements, Plasmids | Phages, Prophages | Phage capsid proteins | Phage head maturation protease | No | Yes | Yes | Yes | Yes |
| Phages, Prophages, Transposable elements, Plasmids | Phages, Prophages | Phage packaging machinery | Phage DNA packaging protein | Yes | No | No | No | No |
| Phages, Prophages, Transposable elements, Plasmids | Phages, Prophages | Phage packaging machinery | Phage portal protein | Yes | No | No | No | No |
| Phages, Prophages, Transposable elements, Plasmids | Phages, Prophages | Phage packaging machinery | Phage portal protein | Yes | No | No | No | No |
| Phages, Prophages, Transposable elements, Plasmids | Phages, Prophages | Phage packaging machinery | Phage terminase ATPase subunit | No | Yes | Yes | Yes | Yes |
| Phages, Prophages, Transposable elements, Plasmids | Phages, Prophages | Phage packaging machinery | Phage terminase, endonuclease subunit | No | Yes | Yes | Yes | Yes |
| Phages, Prophages, Transposable elements, Plasmids | Phages, Prophages | Phage packaging machinery | Phage terminase, small subunit | No | Yes | Yes | Yes | Yes |
| Phages, Prophages, Transposable elements, Plasmids | No sub-category | Integrons | Integron integrase IntI1 | Yes | No | No | No | No |
| Phages, Prophages, Transposable elements, Plasmids | Phages, Prophages | Phage replication | Single stranded DNA-binding protein, phage-associated | Yes | No | No | No | No |
| Phages, Prophages, Transposable elements, Plasmids | Phages, Prophages | Phage tail fiber proteins | Phage tail fiber proteins | Yes | No | No | No | No |
| Phages, Prophages, Transposable elements, Plasmids | Phages, Prophages | Phage tail proteins | Phage minor tail protein | Yes | No | No | No | No |
| Phages, Prophages, Transposable elements, Plasmids | Phages, Prophages | Phage tail proteins | Phage tail assembly | Yes | No | No | No | No |
| Phages, Prophages, Transposable elements, Plasmids | Phages, Prophages | Phage tail proteins | Phage tail assembly protein | Yes | No | No | No | No |
| Phages, Prophages, Transposable elements, Plasmids | Phages, Prophages | Phage tail proteins | Phage tail assembly protein I | Yes | No | No | No | No |
| Phages, Prophages, Transposable elements, Plasmids | Phages, Prophages | Phage tail proteins | Phage tail completion protein | Yes | No | No | No | No |
| Phages, Prophages, Transposable elements, Plasmids | Phages, Prophages | Phage tail proteins | Phage tail length tape measure protein 1 | Yes | No | No | No | No |
| Phages, Prophages, Transposable elements, Plasmids | Phages, Prophages | IbrA and IbrB: co-activators of prophage gene expression | Co-activator of prophage gene expression IbrA, IbrB | Yes | No | No | No | No |
| Phages, Prophages, Transposable elements, Plasmids | Transposable elements | CBSS-203122.1 2.peg.188 | ISPsy4, transposition helper protein | No | Yes | Yes | Yes | Yes |
| Phages, Prophages, Transposable elements, Plasmids | Transposable elements | CBSS-203122.1 2.peg.188 | TniA putative transposase | No | Yes | Yes | Yes | Yes |
| Phages, Prophages, Transposable elements, Plasmids | Transposable elements | CBSS-203122.1 2.peg.188 | TniB NTP-binding protein | No | Yes | Yes | Yes | Yes |
| Phages, Prophages, Transposable elements, Plasmids | Transposable elements | CBSS-203122.1 2.peg.188 | Transposase OrfAB, subunit B | No | Yes | Yes | Yes | Yes |
